# Recapitulating Alzheimer’s disease pathophysiology with a microfluidic neurospheroid-grafted endothelial barrier model

**DOI:** 10.1186/s13041-026-01285-3

**Published:** 2026-03-03

**Authors:** Samuel Chidiebere Uzoechi, Charity Johnson-Campbell, Cody Joseph Badeaux, Sang Su Kwak, Doo Yeon Kim, Yeoheung Yun

**Affiliations:** 1 FIT BEST Laboratory, Department of Chemical, Biological and Bio Engineering, NERVE Center, North Carolina Agricultural and Technical State University, Greensboro, NC 27411 USA; 2 Genetics and Aging Research Unit, Mass General Hospital, Harvard Medical School, Charlestown, MA 02129 USA

**Keywords:** Alzheimer’s disease (AD), endothelial barrier, neurospheroid, microfluidics, endothelial cells (ECs)

## Abstract

**Background:**

Traditional two-dimensional (2D) models do not adequately capture the complex cellular interactions, brain-specific architecture, and progressive pathology of Alzheimer’s disease (AD). Three-dimensional (3D) organoid and microfluidic technologies provide more physiologically relevant platforms for studying AD-associated neurovascular dysfunction.

**Methods:**

We developed a membrane-free microfluidic endothelial barrier model integrated with neurospheroids derived from familial AD (FAD) neural progenitor cells. Human endothelial cells were cultured within perfusable microfluidic channels to establish a vascular-like interface rather than a fully specialized BBB endothelium. Pre-differentiated neurospheroids were grafted into the brain chamber. Endothelial barrier integrity, tight-junction expression, phosphorylated tau (pTau), and Aβ42/Aβ40 production and distribution between compartments were assessed using immunofluorescence imaging and ELISA.

**Results:**

The neurospheroid-grafted endothelial barrier construct captured key AD-associated phenotypes. ReN-AD-D4 models exhibited increased endothelial barrier permeability, reduced ZO-1 expression, and elevated pTau relative to controls. The platform supported endogenous Aβ generation, accumulation, and endothelial-associated deposition at the endothelial barrier. ELISA demonstrated differential Aβ42 and Aβ40 distribution, consistent with isoform-selective behavior reported in AD pathology. Collectively, these results indicate co-occurring neuronal and endothelial barrier alterations within the integrated 3D system.

**Conclusion:**

This microfluidic endothelial barrier–neurospheroid platform enables quantitative assessment of amyloid-β accumulation, spatial distribution, and compartmentalized secretion alongside tau pathology and endothelial barrier integrity changes. Integrating human endothelial monolayers with FAD-derived neurospheroids, the system is scalable and compatible with high-content imaging. Although it does not model BBB-specific transport mechanisms, it provides a robust framework for hypothesis-driven studies of neurovascular interactions and therapeutic screening applications.

**Supplementary Information:**

The online version contains supplementary material available at 10.1186/s13041-026-01285-3.

## Introduction

Alzheimer’s disease (AD) is the most prevalent neurodegenerative disorder, currently affecting over 55 million people worldwide [1], with global cases projected to exceed 150 million by 2050 [2]. In the United States alone, more than 7.2 million individuals aged 65 and older are living with AD in 2025, a number expected to double by 2060, making it the sixth leading cause of death among older adults [3]. The global economic burden of AD and related dementias is staggering, with care costs estimated at over US$1.3 trillion annually [4] and projected to reach a cumulative US$14.5 trillion between 2020 and 2050 [5]. Although disease-modifying treatments are emerging, including anti-amyloid therapies and drug repurposing strategies [6], AD remains biologically complex. It exists in two major forms, the familial form (FAD), which accounts for approximately 1% of cases and is linked to mutations in APP, PSEN1, and PSEN2 genes [7–9], and the sporadic form (SAD), which represents the remaining 99% and results from a combination of genetic and environmental influences [10]. Despite their different origins, both FAD and SAD converge on a common pathological signature, which includes extracellular amyloid beta (Aβ) plaques and intracellular hyperphosphorylated tau (p-tau) tangles [11]. A critical feature of AD progression is dysfunction of the blood-brain barrier (BBB), which exacerbates Aβ and p-tau accumulation, inflammation, and oxidative stress [12, 13].

The BBB is essential for maintaining brain homeostasis and consists primarily of endothelial cells, pericytes, and astrocytic end feet, with microglia closely associated as part of the broader neurovascular unit [14, 15]. Microglia, although not structural components of the BBB, remain functionally integrated through their roles in neuroimmune surveillance and inflammatory signaling relevant to AD [16, 17]. Together, these neurovascular elements preserve barrier integrity, regulate selective transport, and maintain a stable neural environment [18, 19]. BBB dysfunction is a hallmark of neurodegenerative diseases such as AD and is characterized by increased permeability, disrupted tight junctions, and altered interactions among neurovascular cells [18]. Although animal-based in vivo models have contributed important insights into AD pathology, their limited predictive validity has reduced the success of clinical translation [20]. To address these limitations, the Food and Drug Administration (FDA) recently implemented the FDA Modernization Act 2.0, which supports the use of cell-based assays and computational models in preclinical drug development [21]. As a result, there is increasing emphasis on human cell-based in vitro systems as more reliable alternatives to traditional animal models [21]. Traditional two-dimensional (2D) cultures and Transwell co-culture systems offer partial insights but cannot recreate the complex neurovascular environment required to model AD pathophysiology accurately [22].

Recent advances in spheroid and organoid platforms have improved AD modeling by incorporating disease-relevant genetic mutations [23, 24]. Microfluidic tissue chips have also emerged as promising tools for investigating AD mechanisms, integrating induced pluripotent stem cell (iPSC) derived neurons, isogenic organoids, and microvascular models [25, 26]. However, these models remain at an early phase of development and typically lack full coupling of BBB function with patient-derived neuronal pathology. In this study, we introduce a microfluidic three-dimensional (3D) culture platform that incorporates a phase guide-based compartmentalized system enabling the co-culture of familial AD (FAD) neurospheroids integrated with a membrane-free perfused endothelial barrier rather than a fully specialized BBB endothelium [27]. This platform supports perfusion, neurovascular interactions, and biochemical exchange, enabling investigation of AD-related changes in endothelial permeability, Aβ distribution across brain-like and vascular compartments. By combining FAD-specific neuronal pathology with a dynamic endothelial interface, this membrane-free system bridges the gap between existing in vitro models and key features of in vivo neurovascular physiology, offering a scalable and high-throughput framework for assessment of AD-related neurovascular dysfunction and for futuretherapeutic evaluation, without implying specific transport mechanisms.

## Materials and methods

### Cell culture

Primary human umbilical vein endothelial cells (HUVECs, C2519AS; Lonza) [28] were cultured in T-75 flasks (Nunc EasyFlask, Sigma F7552) and used between passages 4 and 6 [25]. Cells were maintained in Endothelial Cell Growth Medium-2 (EGM-2, CC3156; Lonza) supplemented with the EGM-2 BulletKit (CC4176; Lonza), with medium replaced every 3 days. HUVECs were selected as a robust, readily available endothelial source that forms reproducible shear-responsive monolayers in microfluidic channels, while recognizing that they do not fully recapitulate brain microvascular endothelial transporter and tight-junction specialization.

ReNcell VM human neural progenitor cells (ReN-mGAP), provided by Dr. Doo Yeon Kim (Massachusetts General Hospital), were genetically modified using IRES-mediated polycistronic lentiviral vectors containing familial Alzheimer’s disease (FAD) mutations [29]. The vectors encoded human APP carrying the Swedish (K670N/M671L) and London (V717I) mutations, and PSEN1 containing the ΔE9 deletion. Two GFP-tagged ReNcell lines were used: ReN-Ctrl-G10 (control) and ReN-AD-D4 (FAD model). Cells were expanded in T-75 flasks coated with growth factor–reduced Matrigel (Corning 356230) and cultured in DMEM/F-12 medium supplemented with 2% B27 (Thermo Fisher 1704044), 2 µg/mL heparin (StemCell Technologies 07980), 20 ng/mL FGF (StemCell Technologies 78003.1), 20 ng/mL EGF (Sigma-Aldrich E9644), and 1% penicillin–streptomycin–amphotericin B (HyClone SV30010). Cultures were maintained at 37 °C in a humidified incubator with 5% CO₂ [30].

## Pre-differentiation of human 3D neurospheroids

ReN cells were harvested when cultures reached > 80% confluency, following established protocols [29]. Cells were washed with PBS (Corning 21-040-CV) and detached using Accutase (StemCell Technologies 07920), then incubated for 5 min at 37 °C in 5% CO₂. The cell suspension was counted using 0.4% Trypan Blue (Thermo Fisher 15250061) to assess viability. Approximately 20,000 cells were seeded into each well of ultra-low-attachment 96-well round-bottom plates (Sigma-Millipore CLS4591) to generate neurospheroids.

Neurospheroids were maintained in proliferation medium for 3 days. Neuronal differentiation was initiated by removing EGF and FGF from the culture medium. Media were replaced every 3 days for at least 5 weeks to allow neurospheroid maturation before grafting into the vascular compartment of the microfluidic device [31].

## Microfluidic neurospheroid-grafted endothelial barrier tissue construct

**Fig. 1:**
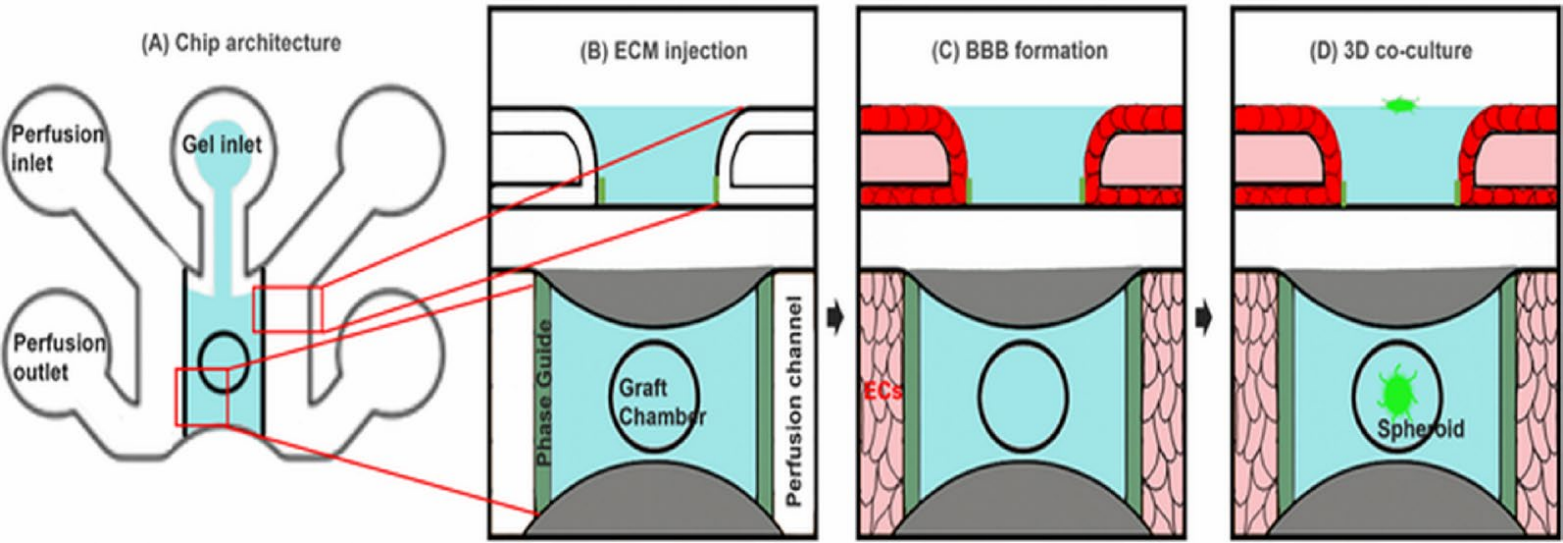
Sequence of steps to the generation of a 3D neurospheroid-grafted BBB construct. **A** Schematic overview of the microfluidic chip architecture, which enables multiplexed experiments with integrated gel lane, perfusion channels, and observation windows, **B** the graft chamber is filled with Collagen I gel (depicted in blue) via the gel inlet, **C** Formation of a functional perfused endothelial barrier structure: Human endothelial cells are seeded into the perfusion channel adjacent to a collagen I matrix, forming a lumenized vessel under gravity-driven bidirectional flow, **D** Co-culture of pre-differentiated 3D human neurospheroids and the endothelial cells. Neurospheroid cells are grafted onto the graft chamber, allowing interaction with the endothelial barrier via the shared matrix interface

The OrganoPlate Graft (MIMETAS BV, USA) was used to generate the perfused endothelial barrier integrated neurospheroid construct (referred to throughout as an endothelial barrier model rather than a fully specialized BBB to reflect the molecular profile of the HUVEC-based system). The platform contains 64 independent culture chips, each with one gel inlet, two perfusion channels, and a central graft chamber (Fig. 1A).

The extracellular matrix was prepared using a collagen solution composed of Type I collagen, HEPES buffer, and sodium bicarbonate at an 8:1:1 ratio (Corning 354249, 9.48 mg/mL; Thermo Fisher 15630-080). The collagen mixture was introduced into the gel inlet and polymerized for 15 min at 37 °C in 5% CO_2_ [32]. Hank’s Balanced Salt Solution (HBSS) was added to the gel inlet after polymerization to prevent dehydration (Fig. 1B).

Endothelial cells were detached using TrypLE Express (Thermo Fisher 12605010), resuspended at 10,000 cells/µL, and seeded into the perfusion channels. The plate was placed on an interval rocker alternating between + 14° and − 14° every 8 min to generate bidirectional flow. Cultures were maintained at 37 °C in 5% CO_2_, and medium was replaced every 2 days. A continuous endothelial barrier is typically formed by day 7 (Fig. 1C).

Pre-differentiated neurospheroids derived from ReNcell VM lines (ReN-Ctrl-G10 and ReN-AD-D4) were transferred into the graft chamber. After grafting, a 75% and 25% mixture of endothelial complete medium and ReN differentiation medium was added to the perfusion channels. The neurospheroids and endothelial monolayers were co-cultured under dynamic flow conditions to support barrier formation, cellular interaction, and integration of the neurospheroid with the endothelial interface (Fig. 1D).

## Barrier integrity assay, and apparent permeability calculation

Barrier integrity was assessed in the OrganoGraft platform by quantifying the transport of FITC-dextran across the endothelial barrier [25]. The perfusion channels were rinsed with EGM-2 medium, after which 0.5 mg/mL FITC-dextran (40 kDa) prepared in complete EGM-2 was introduced into the channels. A total of 20 µL of non-fluorescent basal medium was added to the gel inlet, and 40 µL and 20 µL of the FITC-dextran solution were added to the media inlet and outlet of the perfusion lane, respectively.

Live imaging was conducted using an EVOS fluorescence microscope equipped with an on-stage incubator. Time-lapse images were collected every 30 s for 10 min. Fluorescence intensities from the donor channel and receiver compartment were quantified using Fiji (Version 1.54i, March 2024) to generate normalized concentration profiles over time.

Physical constants were as follows: donor volume (V_D_) = 0.00079 cm³, receiver volume (V_R_) = 0.0036 cm³, and barrier surface area (A) = 0.01 cm^2^. The receiver concentration data C_R_(t) were fitted to the exact model equation as implemented previously for OrganoPlate [33] to obtain the apparent permeability coefficient.

1 $$ \:CR\left( t \right) = \left\langle {{\mathrm{C}}\left( {\mathrm{t}} \right)} \right\rangle (1 - {\mathrm{exp}}[ - k_{p} (t - t_{{j - 1}} )]) $$

where the rate constant *k*_*p*_, incorporates the apparent permeability (*P*_*app*_):

2 $$ k_{p} = \left. {\left( {\frac{1}{{V_{D} }}} \right. + \frac{1}{{V_{R} }}} \right)P_{{app}} A $$

Nonlinear regression was used to estimate k_p_ and compute P_app_ by minimizing the deviation between modeled and experimental fluorescence data.

## Soluble Aβ quantification

Soluble Aβ was quantified in the brain parenchyma–like fluid (BPF) within the neurospheroid compartment and in the blood-like fluid (BF) collected from the perfusion channels. Concentrations of Aβ_1-40_ and Aβ_1-42_ were measured using a Human β-Amyloid (1–40 and 1–42) ELISA kit (Wako Chemicals 290-62601, Osaka, Japan) [34].

Culture medium samples were collected every 2 days for a total of 12 days and stored at − 20 °C until analysis. Before processing, frozen supernatants were thawed at 4 °C, and total protein concentrations were measured using the Pierce BCA Protein Assay Kit (Thermo Scientific 23225). ELISA was performed using equal protein amounts following the manufacturer’s instructions. Absorbance was read at 450 nm using a CLARIOstar Plus spectrophotometer (BMG LabTech, Germany).

### Quantification of neurospheroid diameter and neurite outgrowth

Fluorescence images of the tissue constructs within the microfluidic device were acquired using a fluorescence microscope. Image analysis was performed using MATLAB to quantify neurospheroid diameter and neurite outgrowth.

A polygonal region of interest was manually drawn around each neurospheroid using the “drawpolygon” function. The enclosed area was calculated, and the neurospheroid diameter was derived using the following equation, in which A represents the measured area:

3 $$\:{D}_{iameter}=2\:\mathrm{x}\sqrt{\frac{\mathrm{A}}{{\uppi\:}}}$$

To quantify neurite outgrowth, the “drawpolygon” function was used to manually outline individual neurites and measure their diameters and spatial extent. A second polygon was drawn around the neurospheroid core as observed at day zero. The area of the day zero neurospheroid was subtracted from the total projected area measured on day eight to isolate the neurite mass from the cell body and determine the net outgrowth area.

## Immunocytochemistry

The neurospheroid–endothelial barrier constructs cultured for 12 days in the microfluidic device were fixed with 4% paraformaldehyde (PFA; Thermo Fisher J19943-K2) overnight at room temperature. After fixation, samples were permeabilized with 0.5% Triton X-100 (Thermo Fisher EP151-100) for 20 min at room temperature, followed by a 5 min wash in Tris-buffered saline containing 0.1% Tween-20 (TBS-T; Invitrogen AM9856; Sigma Aldrich P9416).

Constructs were blocked with 4% skim milk (Millipore AM1082963) prepared in TBS-T for 24 h at 4 °C. Primary antibody incubation was performed for 24 h at 4 °C in a buffer containing 50 mM Tris-HCl at pH 7.4 (Thermo Fisher J22638.AE), 0.1% Tween-20, 4% donkey serum (Southern Biotech 0030 − 01), 1% bovine serum albumin (Sigma Aldrich A8412), 0.1% gelatin (StemCell Technologies 07903), and 0.3 M glycine (Thermo Scientific 036435.30) [29].

The following primary antibodies were used: MAP2 (1:2500; Thermo Fisher PA1-10005), phospho-Tau (Ser202/Thr205) (1:250; Thermo Fisher MN1020), Aβ₄₂ and Aβ₄₀ (1:150; Thermo Fisher MA5-36246 and 36-6900), β-tubulin III (1:500; Sigma Aldrich AB9354), GFAP IgG1 Alexa Fluor 594 (1:500; Invitrogen A21295), Alexa Fluor 594 anti-CD31 (1:500; Abcam EPR3094), and Alexa Fluor 488 anti-ZO-1 (1:250; Invitrogen 339188).

Secondary antibodies used were Alexa Fluor 594 (Invitrogen A21203 or A21207) and Alexa Fluor 647 (Invitrogen A78952 or A32787), each at 1:250. Antibodies were added to the grafting chamber and incubated overnight at 4 °C. After washing, Hoechst 33,342 (1:1000; Invitrogen H3570) was applied for 15 min for nuclear staining.

Fluorescence images were acquired using a ZEISS LSM 710 two-photon confocal microscope, and data were processed using ZEN Blue software.

## Image analysis

Images were acquired using a ZEISS LSM 710 two-photon confocal microscope and processed with ZEN Blue software. Morphological analysis of ZO-1 and quantification of F-actin and CD31 fluorescence intensity in HUVECs were performed using Fiji (ImageJ) software (Version 1.54i, March 2024). Using a consistent threshold across all images, F-actin, ZO-1, and CD31 signals were measured, and average values were calculated (*n* = 4 for conditions).

### Statistical analysis

Statistical analysis was performed using GraphPad Prism software (version 8.0, GraphPad Software, San Diego, CA, USA). Data are presented as mean ± standard deviation (SD). Comparisons between two independent groups were performed using two-tailed Welch’s *t*-test. For datasets involving more than two groups, ordinary one-way analysis of variance (ANOVA) was used, followed by Šídák’s multiple-comparisons test. For analyses involving multiple molecular markers across experimental conditions, two-way ANOVA with post hoc multiple-comparisons testing was applied. Multiple endpoints were analyzed as predefined, biologically distinct outcomes in hypothesis-driven comparisons; therefore, no global correction across figures was applied. Exact *P* values are reported, and statistical significance was defined as *p* < 0.05 unless otherwise stated.

## Results

### Microfluidic neurospheroid-grafted endothelial barrier tissue construction

**Fig. 2:**
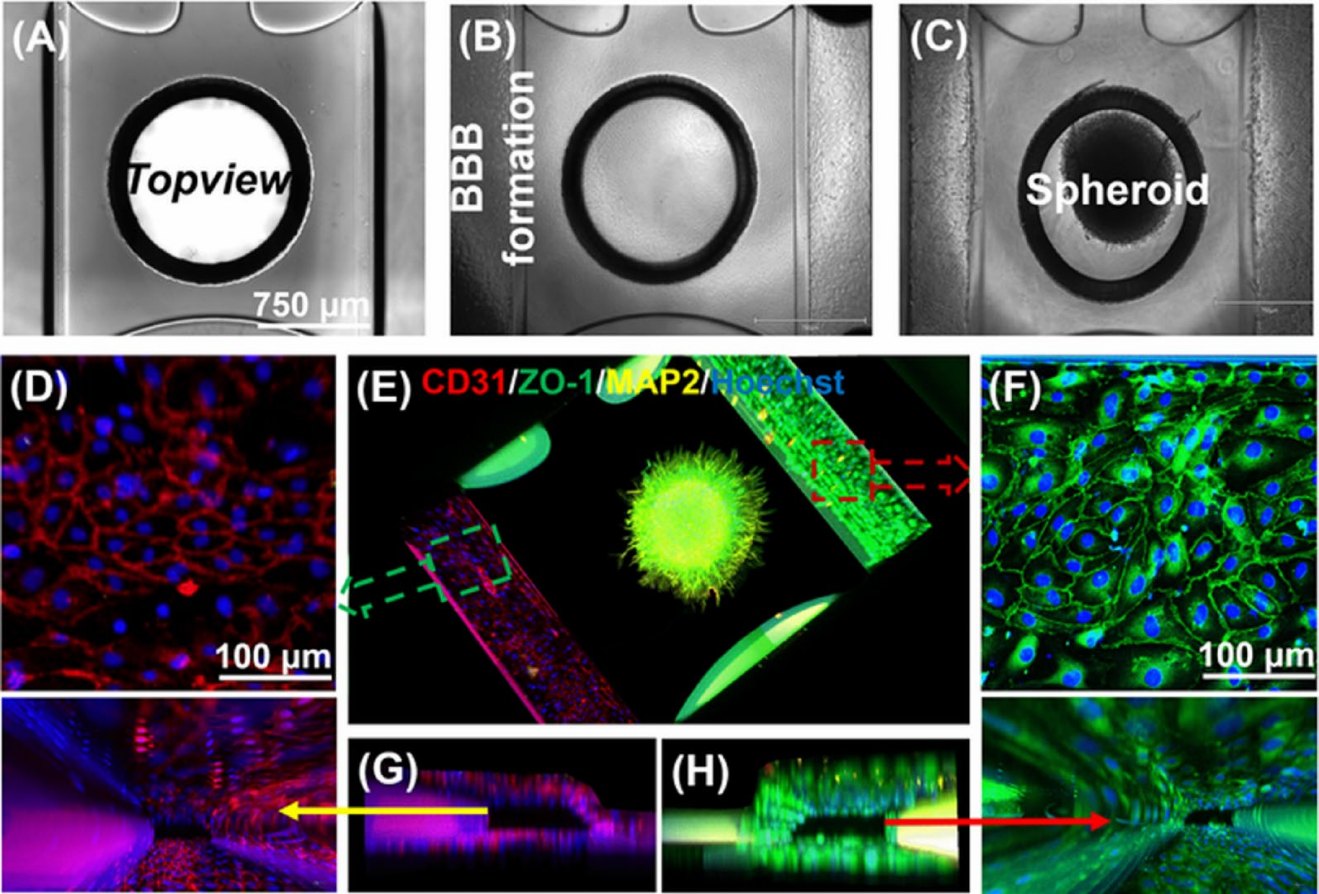
Illustration of the development of a reproducible 3D neurospheroid-grafted endothelial barrier model using a microfluidic device. **A**–**C** show the experimental workflow, including ECM injection, endothelial barrier formation and neurospheroid placement, respectively, **D** shows CD31 (red) for endothelial cell adhesion marker, **E** depicts neurospheroids in the brain chamber alongside tubular endothelial barrier structures, **F** presents ZO-1 (green) labeling tight junctions, and **G, H** confirms lumen formation and apical perfusion. Scale bar: 100 μm

Figure 2 shows the generation of the microfluidic neurospheroid-grafted endothelial barrier construct and representative images of the assembled system. Endothelial cells seeded in both perfusion channels formed a continuous tubular structure within 7 days at a seeding density of 10,000 cells per channel (Figs. 2A–B). Neurospheroid density was evaluated between 10,000 and 50,000 cells, and 10,000-cell aggregates produced stable spheroids with diameters of 300–400 μm (Supplementary Fig. S1).

Pre-differentiated ReN-Ctrl-G10 and ReN-AD-D4 neurospheroids were introduced into the graft chamber of the microfluidic device (Fig. 2C). Media composition was optimized such that endothelial cells and neurospheroids received 75% and 25% of their respective media in the perfusion channels, with the reverse ratio applied in the graft chamber. Cultures were maintained on a rocker cycling between + 14° and − 14° every 8 min to generate bidirectional flow and shear stress levels of approximately 0.5–5 dyne/cm^2^ [26, 32].

Under these dynamic conditions, the co-culture remained structurally stable and supported the formation of an integrated 3D construct. Immunofluorescence imaging demonstrated the presence of neurospheroids (MAP2, yellow) and endothelial structures expressing CD31 (red) and ZO-1 (green) (Figs. 2D–F). A clear lumen architecture was observed, and the membrane-free endothelial barrier structure remained intact for more than 19 days (Figs. 2G–H).

### Neurospheroid-spreading in the microfluidic device

**Fig. 3:**
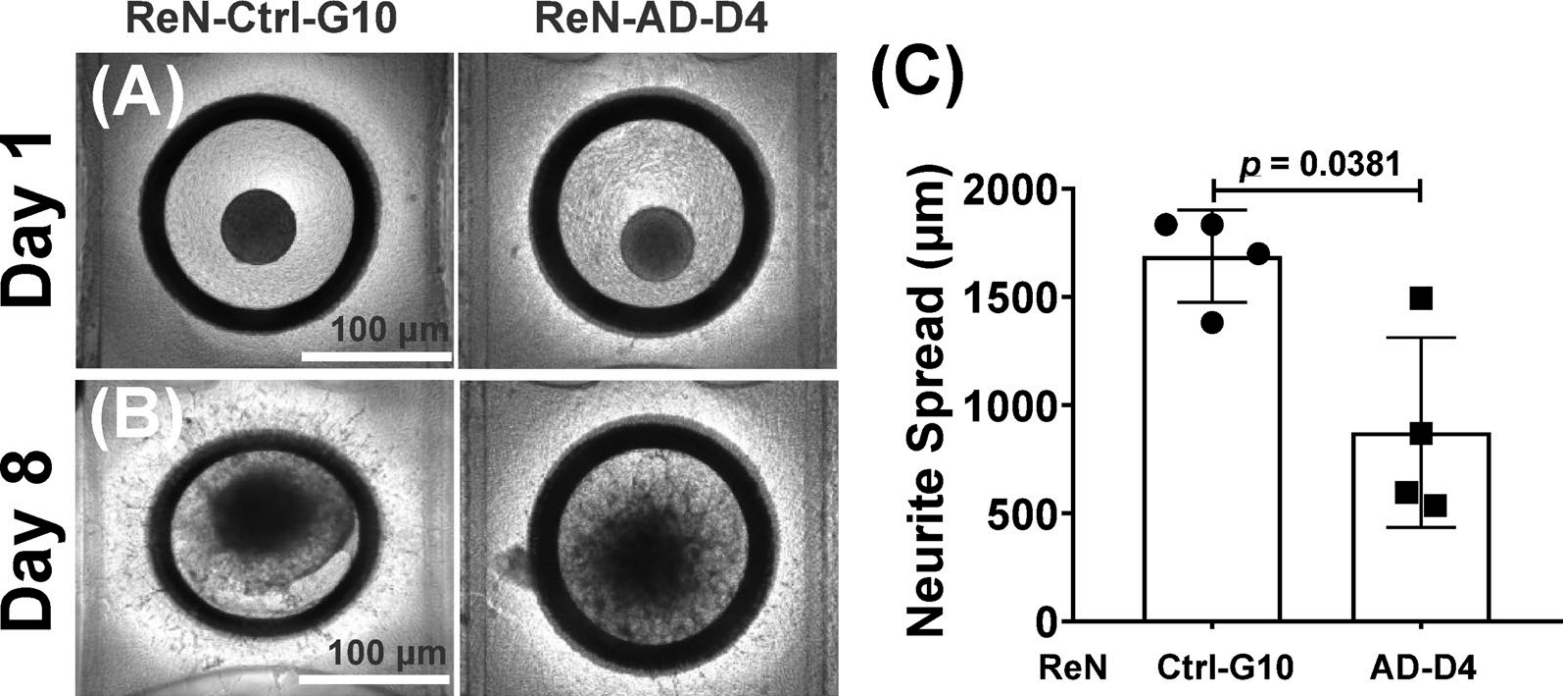
Microfluidic neurospheroid culture reveals reduced neurite outgrowth in FAD-derived neural spheroids. Representative brightfield images and quantitative analysis of ReN-Ctrl-G10 and ReN-AD-D4 neurospheroids cultured in a microfluidic device. **A**, **B** Brightfield images of neurospheroids on Day 1 and Day 8 (scale bar: 100 μm), **C** quantification of neurite outgrowth in ReN-Ctrl-G10 and ReN-AD-D4 neurospheroids. Bars represent mean ± SD from *n* = 4 independent experiments; dots indicate individual experiments (each dot represents the average of 4 neurospheroids). Statistical significance was determined using two-tailed Welch’s t-test to account for unequal variance (*p* = 0.0381)

Pre-differentiated ReN-Ctrl-G10 and ReN-AD-D4 neurospheroids (5 weeks) were implanted into the graft chamber of the microfluidic device (Fig. 3A–B). ReN-Ctrl-G10 neurospheroids displayed extensive neurite extension, whereas ReN-AD-D4 neurospheroids exhibited markedly reduced outgrowth. Quantification showed that ReN-AD-D4 neurites reached a mean length of 873.3 ± 36.2 μm compared with 1687.6 ± 30.8 μm in ReN-Ctrl-G10, corresponding to a 0.52-fold reduction (*p* = 0.0381; Fig. 3C). These findings demonstrate a substantial decrease in neurite extension in the AD-derived neurospheroids within the microfluidic platform.

### AD-specific phenotypic expression of the neurospheroid in the microfluidic device

**Fig. 4:**
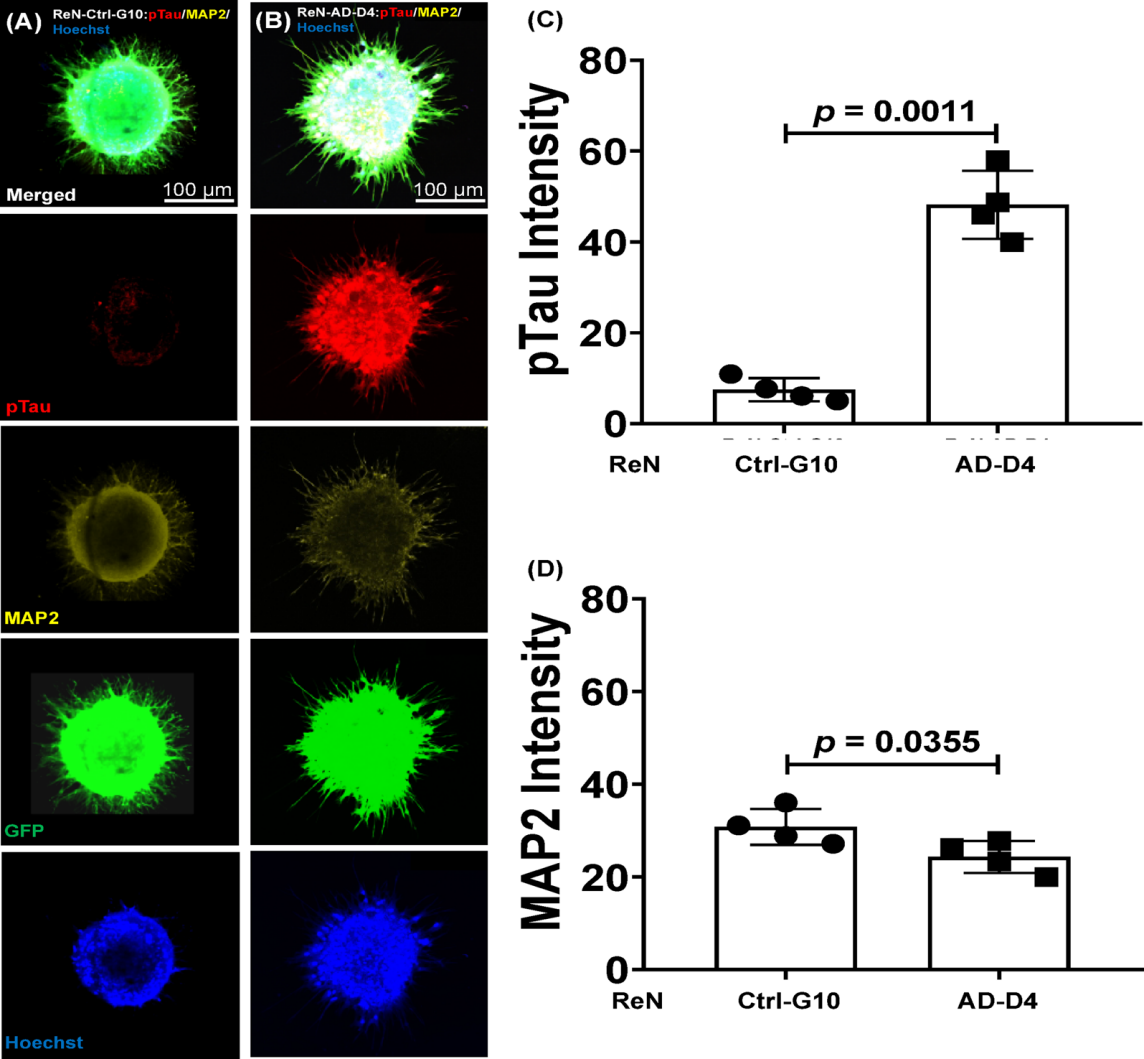
Phosphorylated tau and MAP2 distribution in control and familial Alzheimer’s disease neurospheroids cultured within the microfluidic device. **A**, **B**) Representative confocal images of ReN-Ctrl-G10 and ReN-AD-D4 neurospheroids immunostained for phosphorylated tau (pTau, red) and MAP2 (yellow), with nuclei counterstained using Hoechst (blue). Scale bars, 100 μm, **C**, **D** Qquantitative analysis of fluorescence intensity indicates higher pTau signal in ReN-AD-D4 neurospheroids relative to ReN-Ctrl-G10. MAP2 fluorescence intensity also differs between groups. Bars represent mean ± SD from *n* = 4 independent experiments, with 4 neurospheroids analyzed per condition. Statistical analysis was performed using a two-tailed Welch’s t-test, yielding exact *P* values of 0.0011 for pTau and 0.0355 for MAP2

Immunofluorescence staining showed MAP2 expression and phosphorylated Tau (pTau; Ser202/Thr205) in both ReN-Ctrl-G10 and ReN-AD-D4 neurospheroids cultured in the microfluidic device (Fig. 4A–B). After 8 days of differentiation, ReN-AD-D4 neurospheroids displayed a 7.6-fold increase in pTau intensity relative to ReN-Ctrl-G10 (*p* = 0.0011; Fig. 4C). MAP2 expression was reduced in ReN-AD-D4 neurospheroids, showing a 0.8-fold decrease compared with ReN-Ctrl-G10 (*p* = 0.0355; Fig. 4D).

For reference, neurospheroids were also analyzed in Matrigel, and expression of β3-tubulin, MAP2, pTau, and GFAP is shown in Supplementary Figs. S2 and S3. Both neurospheroid types expressed β3-tubulin, MAP2, and GFAP, whereas pTau levels remained higher in ReN-AD-D4, showing a 5-fold increase compared with ReN-Ctrl-G10 (Supplementary Fig. S2). These supplementary data provide context for phenotype validation, while the primary analyses in this study focus on constructs generated using collagen I.

### AD-associated Aβ burden coincides with endothelial barrier breakdown

**Fig. 5:**
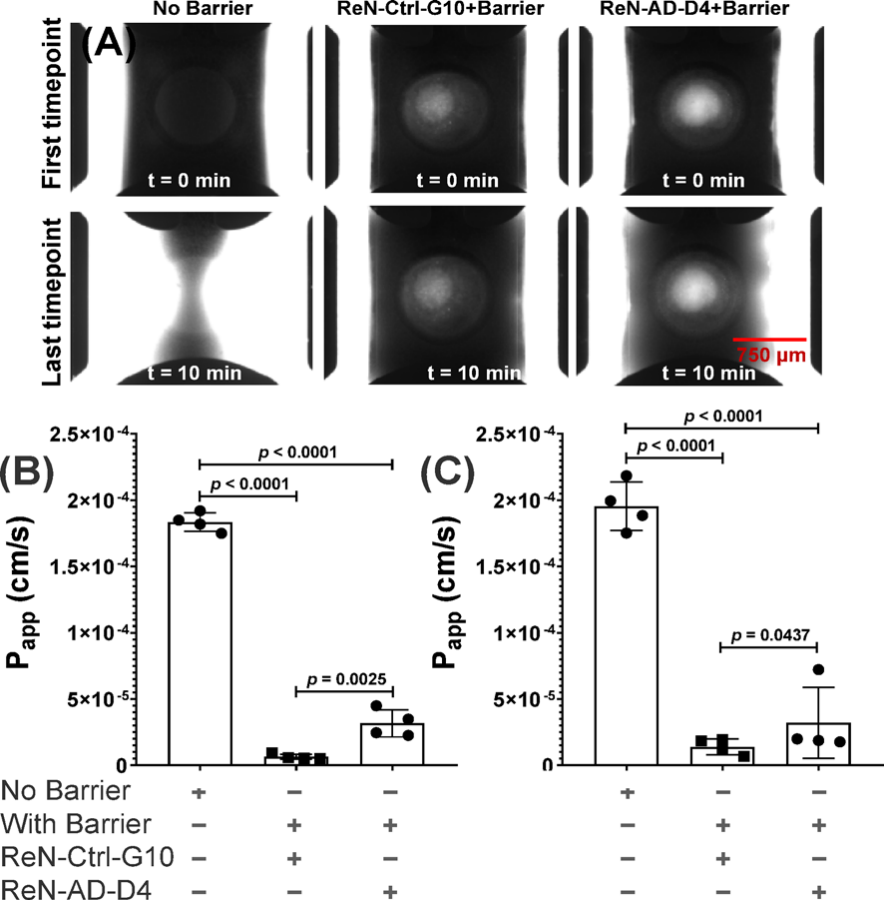
FITC–dextran permeability across neurospheroid-grafted microfluidic endothelial barriers. **A** Representative fluorescence images showing the distribution of 40 kDa FITC–dextran at the initial (t = 0 min) and final (t = 10 min) time points in microfluidic devices containing ReN-Ctrl-G10 or ReN-AD-D4 neurospheroids, with or without an endothelial barrier. Images illustrate differences in tracer distribution over time. Scale bar, 750 μm, **B** Quantification of apparent permeability (P_app_) for 40 kDa FITC–dextran measured at = day 4, **C** quantification of apparent permeability (P_app_) for 40 kDa FITC–dextran measured at day 8. “No Barrier” denotes devices lacking an endothelial lining. Permeability was quantified across the endothelial-lined vascular channels in devices containing grafted neurospheroids. In the annotation, **‘+’** denotes the presence and **‘–’** denotes the absence of the indicated barrier or cell type. Data are presented as mean ± SD from *n* = 4 independent experiments, with 8 microfluidic vascular channels analyzed per condition. Individual data points represent independent experiments, with each point corresponding to the average of 4 microfluidic chips. Statistical analysis was performed using ordinary one-way ANOVA followed by post hoc multiple-comparisons testing. Exact *P* values are reported on the graph

**Fig. 6:**
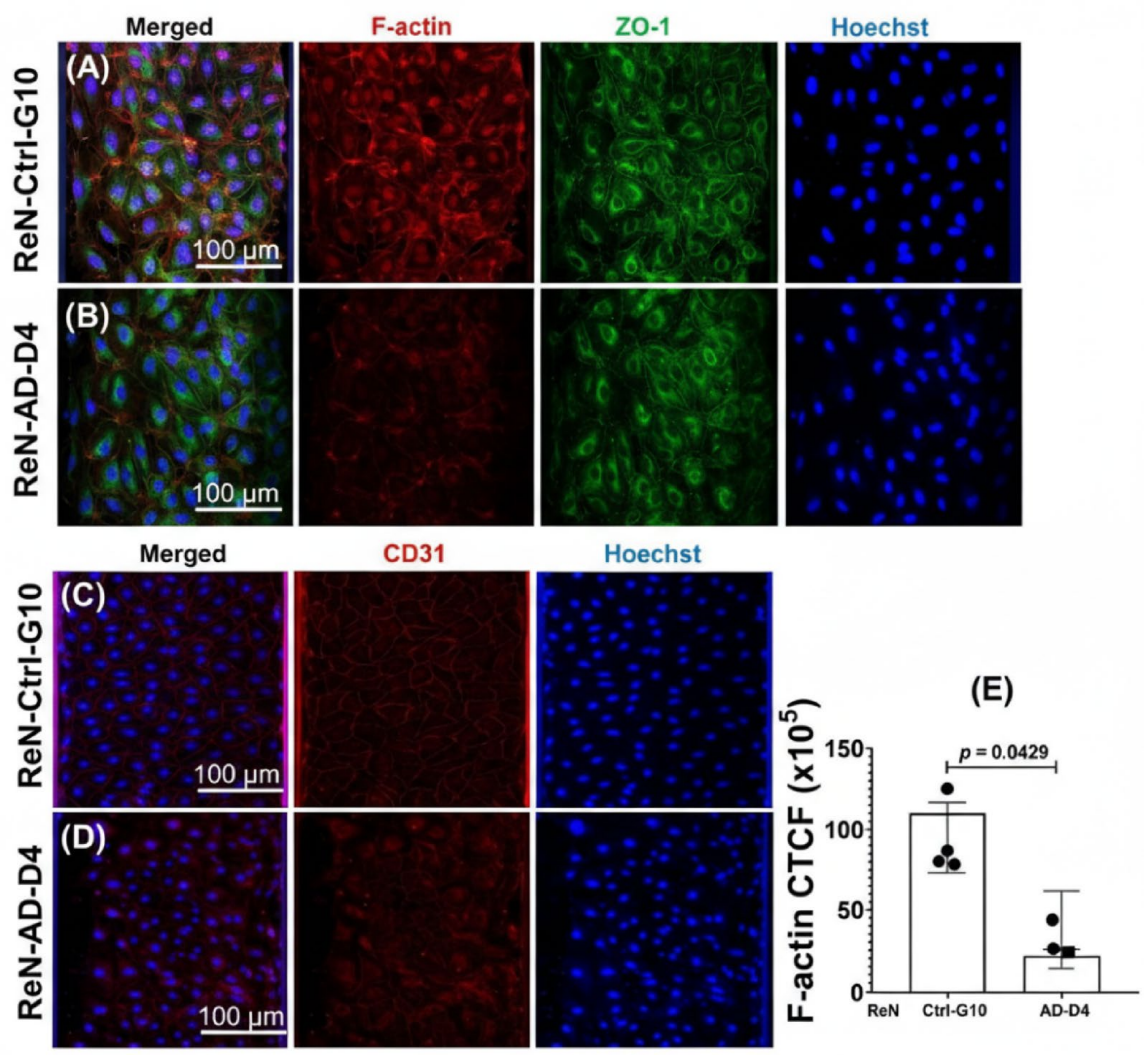
Immunofluorescence staining and fluorescence quantification of endothelial monolayers cultured in microfluidic channels. **A**, **B** Representative images of F-actin (red), ZO-1 (green), and Hoechst (blue) staining of endothelial monolayers cultured under microfluidic conditions. **C**, **D** Representative images of CD31 (red) and Hoechst (blue) staining acquired from the same microfluidic platform. Scale bars = 100 μm, **E** corrected total cell fluorescence (CTCF) for F-actin quantified from thresholded regions of interest (ROIs) in Fiji/ImageJ using identical exposure and analysis settings across groups. All analyses were performed on the basal plane of the microfluidic vascular channels. Data represent mean ± SD from *n* = 4 independent experiments, with 4 microfluidic channels analyzed per condition. Statistical significance was determined using Welch’s t-test to account for unequal variance, with *p* = 0.0429 relative to ReN-Ctrl-G10

Permeability was evaluated in ReN-Ctrl-G10 and ReN-AD-D4 neurospheroid-grafted endothelial constructs to assess barrier function in the presence of AD-associated pathology (Figs. 5 and 6). FITC-dextran assays showed higher permeability in ReN-AD-D4 constructs compared with ReN-Ctrl-G10 (Fig. 5A, B). The apparent permeability (P_app_) for ReN-Ctrl-G10 averaged 7.27 × 10^−6^ cm/s across days 4 and 8, whereas ReN-AD-D4 constructs showed a mean P_app_ of 3.94 × 10⁻⁵ cm/s over the same period. In the absence of an endothelial barrier (No barrier condition), P_app_ values were substantially higher, averaging ~ 1.81 × 10^−4^ cm/s, confirming that the presence of the endothelial barrier markedly reduced permeability (Fig. 5B, C).

### Aβ-Induced disruption of cytoskeletal, tight-junction, and endothelial membrane proteins

Fluorescence images were acquired from the basal plane of the microfluidic chips, corresponding to the endothelial monolayer attached to the channel surface (Figs. 6A–D). In ReN-Ctrl-G10 constructs, F-actin, ZO-1, and CD31 exhibited continuous and uniform staining across the monolayer (Fig. 6A and C). In contrast, ReN-AD-D4 endothelial layers showed pronounced structural disruption, including fragmented F-actin fibers and discontinuous, irregular ZO-1 and CD31 junctional labeling (Fig. 6B and D). These staining patterns indicate altered cytoskeletal organization and loss of tight-junction continuity in the presence of Aβ-associated AD phenotypes.

**Fig. 7:**
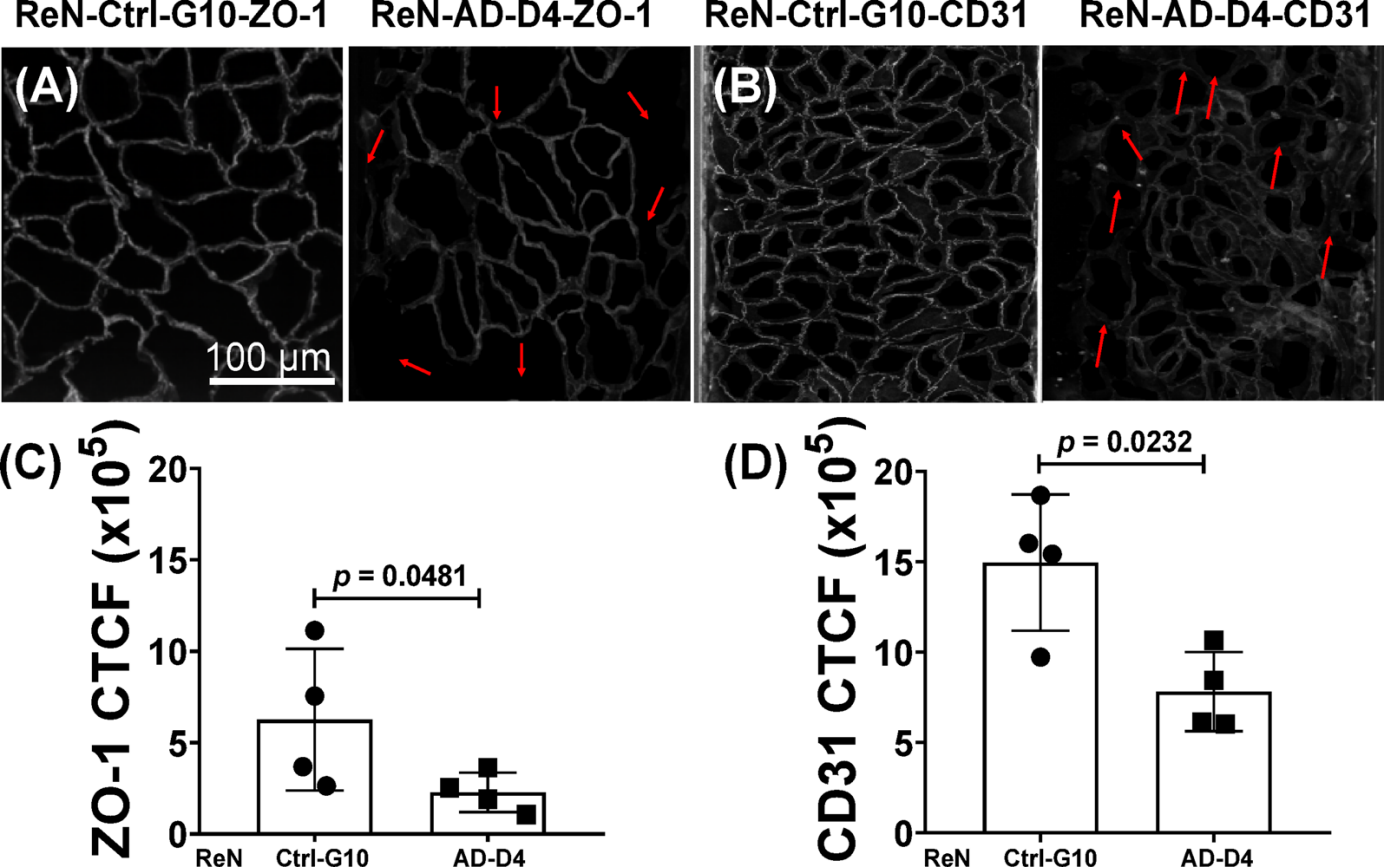
Organization of ZO-1 and CD31 in endothelial monolayers cultured within microfluidic vascular channels. (A–B) Representative regions of interest (ROIs) showing ZO-1 (junctional) and CD31 (membrane) staining in control (ReN-Ctrl-G10) and Aβ-treated (ReN-AD-D4) endothelial monolayers. Arrows indicate regions of disrupted ZO-1 junctional continuity and altered CD31 membrane organization. Scale bar, 100 μm. **C**, **D** Quantification of corrected total cell fluorescence (CTCF) for ZO-1 **C** and CD31 **D** measured from thresholded ROIs using Fiji/ImageJ with identical exposure, thresholding, and analysis parameters across all conditions. Analyses were performed on the basal plane of the microfluidic vascular channels. Data are presented as mean ± SD from *n* = 4 independent experiments, with 4 microfluidic channels analyzed per condition. Statistical analysis was performed using two-tailed Welch’s t-test. Exact *p* values for each comparison are reported directly on the graphs.

Quantitative analysis using corrected total cell fluorescence (CTCF) showed a 36% reduction in F-actin intensity (0.64-fold) in ReN-AD-D4 monolayers compared with ReN-Ctrl-G10 **(**Fig. 6E). Representative regions of interest (ROIs) for ZO-1 and CD31 are shown in Figs. 7A–B, illustrating continuous ZO-1 borders and uniform CD31 staining in ReN-Ctrl-G10, in contrast to fragmented ZO-1 junctions and altered CD31 membrane labeling in ReN-AD-D4. CTCF measurements indicated a 64% reduction in ZO-1 expression (0.36-fold) (Fig. 7C) and a 48% reduction in CD31 intensity (0.52-fold) in ReN-AD-D4 monolayers (Fig. 7D).

### Aβ induces a structural–functional collapse of endothelial barrier integrity

**Fig. 8:**
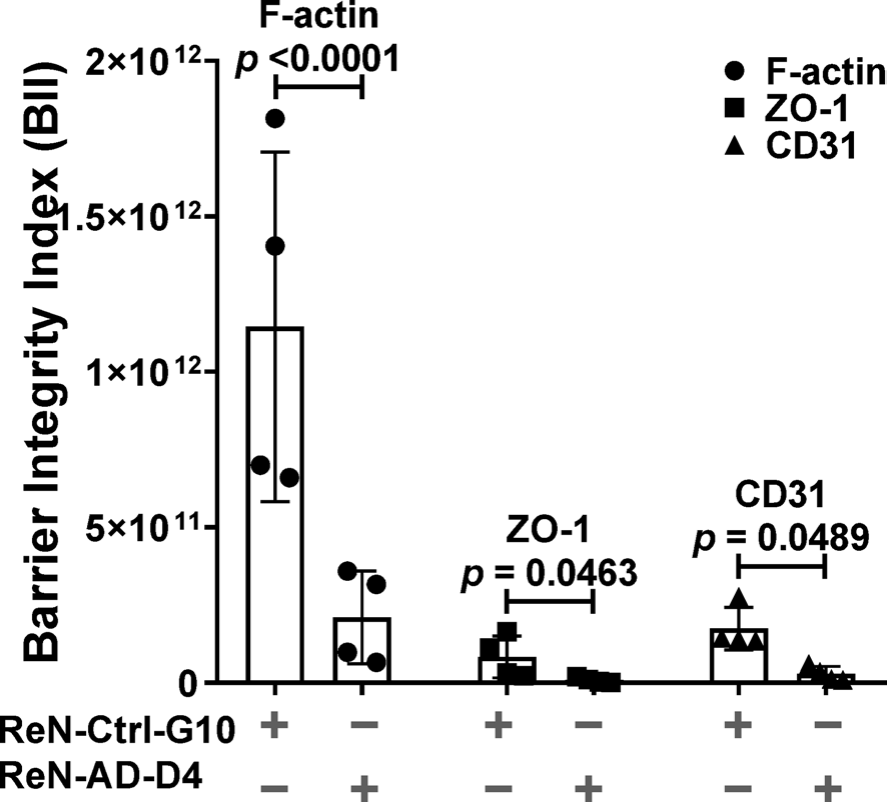
Barrier Integrity Index (BII) integrates structural and permeability measurements in endothelial monolayers cultured within microfluidic vascular channels. Bar graphs show the Barrier Integrity Index (BII), calculated as the ratio of corrected total cell fluorescence (CTCF) to apparent permeability (P_app_), for F-actin, ZO-1, and CD31 in control (ReN-Ctrl-G10) and Aβ-treated (ReN-AD-D4) endothelial monolayers. Data are presented as mean ± SD from *n* = 4 independent experiments, with 4 microfluidic channels analyzed per condition. Statistical analysis was performed using two-way ANOVA with post hoc multiple-comparisons testing. Exact *P* values for each comparison are reported directly on the graphs

A unified structural–functional assessment was performed by pairing corrected total cell fluorescence (CTCF) values for F-actin, ZO-1, and CD31 with their corresponding apparent permeability (P_app_) values from matched microfluidic channels. A Barrier Integrity Index (BII), defined as CTCF/P_app_, was calculated for each replicate (Fig. 8). Using this metric, Aβ-associated conditions showed reduced barrier integrity across all markers. F-actin BII decreased 5.5-fold compared with ReN-Ctrl-G10 monolayers (*p* < 0.0001), CD31 BII decreased 6.1-fold (*p* = 0.0489), and ZO-1 showed the largest decrease at 9.2-fold (*p* = 0.0463). These combined structural and permeability measurements indicate substantial impairment of endothelial barrier properties in the presence of Aβ **(**Fig. 8).

### Aβ buildup and ZO-1 loss demonstrate endothelial barrier dysfunction in the AD model

**Fig. 9:**
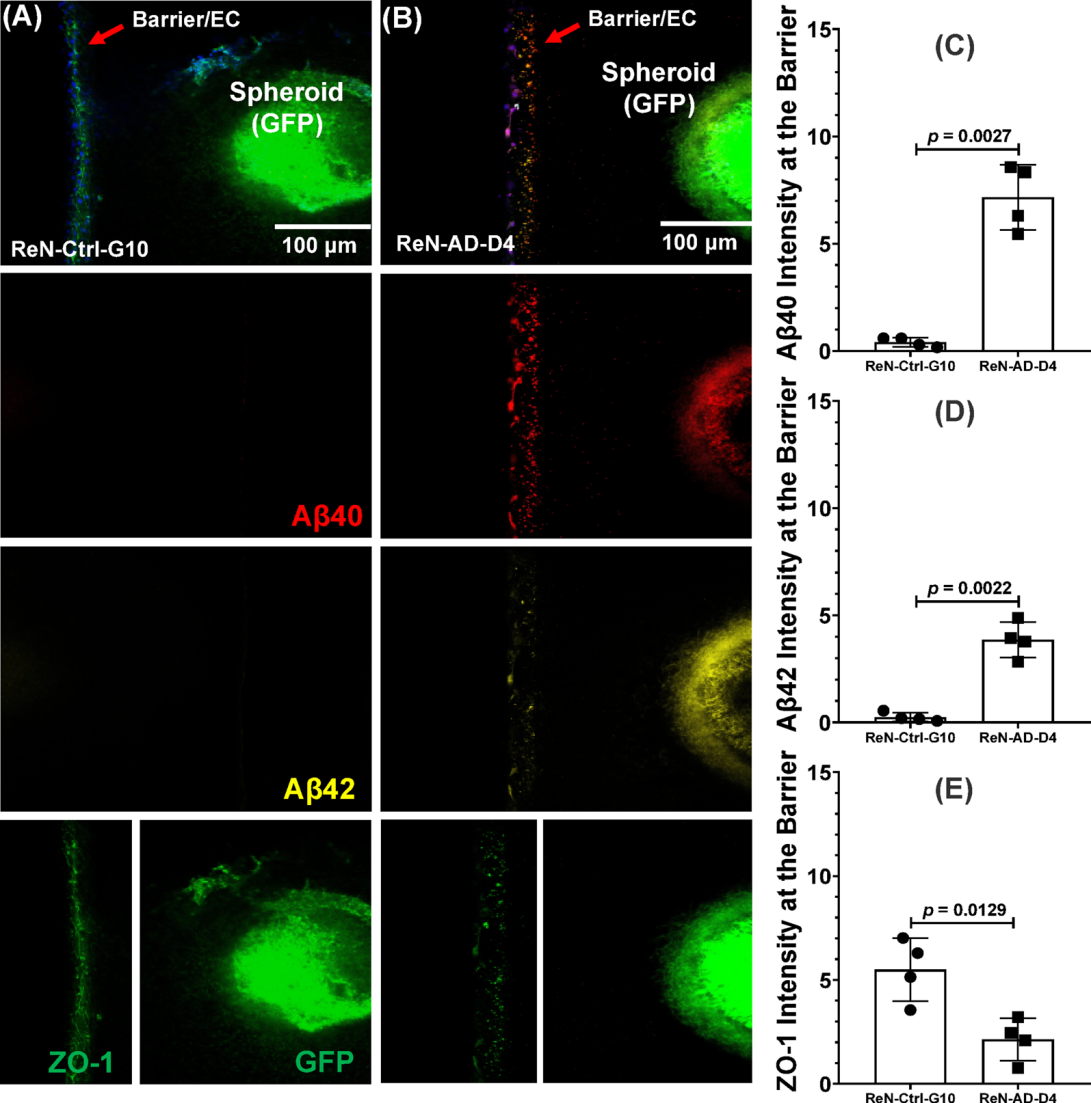
Amyloid-β (Aβ) accumulation in neurospheroid-grafted endothelial barriers within a microfluidic microenvironment. (**A**–**B**) Representative images showing GFP-labeled neurospheroids, endothelial cell (EC) barrier, Aβ40 (red), Aβ42 (yellow), and ZO-1 (green) in ReN-Ctrl-G10 and ReN-AD-D4 constructs after 12 days of culture. Arrows indicate the endothelial barrier region. Scale bar, 100 μm. (**C–E**) Quantification of fluorescence intensity for Aβ40 (**C**), Aβ42 (**D**), and ZO-1 (**E**) measured from defined regions of interest using identical acquisition and analysis parameters across all conditions. Data are presented as mean ± SD from *n* = 4 independent experiments, with 8 microfluidic chips analyzed per condition. Statistical analysis was performed using two-tailed Welch’s *t*-test. Exact *p* values for each comparison are reported directly on the graphs.

Figure 9A–B show immunostaining and quantification of Aβ40 and Aβ42 deposition on the endothelial barrier and residual amyloid within neurospheroids. On the endothelial surface, Aβ40 intensities measured 0.21 ± 0.19 in the ReN-Ctrl-G10 model and 6.96 ± 4.68 in ReN-AD-D4 (p = 0.0027; Fig. 9C). Aβ42 deposition showed a similar pattern, with intensities of 0.05 ± 0.38 in ReN-Ctrl-G10 and 3.66 ± 2.65 in ReN-AD-D4 (p = 0.0022; Fig. 9D). Overall, Aβ40 accumulation was approximately twice that of Aβ42. ZO-1 expression in endothelial cells was also reduced in the presence of Aβ, decreasing from 5.30 ± 2.99 in ReN-Ctrl-G10 to 1.93 ± 1.02 in ReN-AD-D4 (p < 0.0129; Fig. 9E). These measurements show increased amyloid deposition and reduced tight-junction labeling in constructs containing AD-derived neurospheroids.

### AD-associated Aβ dynamics in a neurospheroid-grafted endothelial barrier construct

**Fig. 10:**
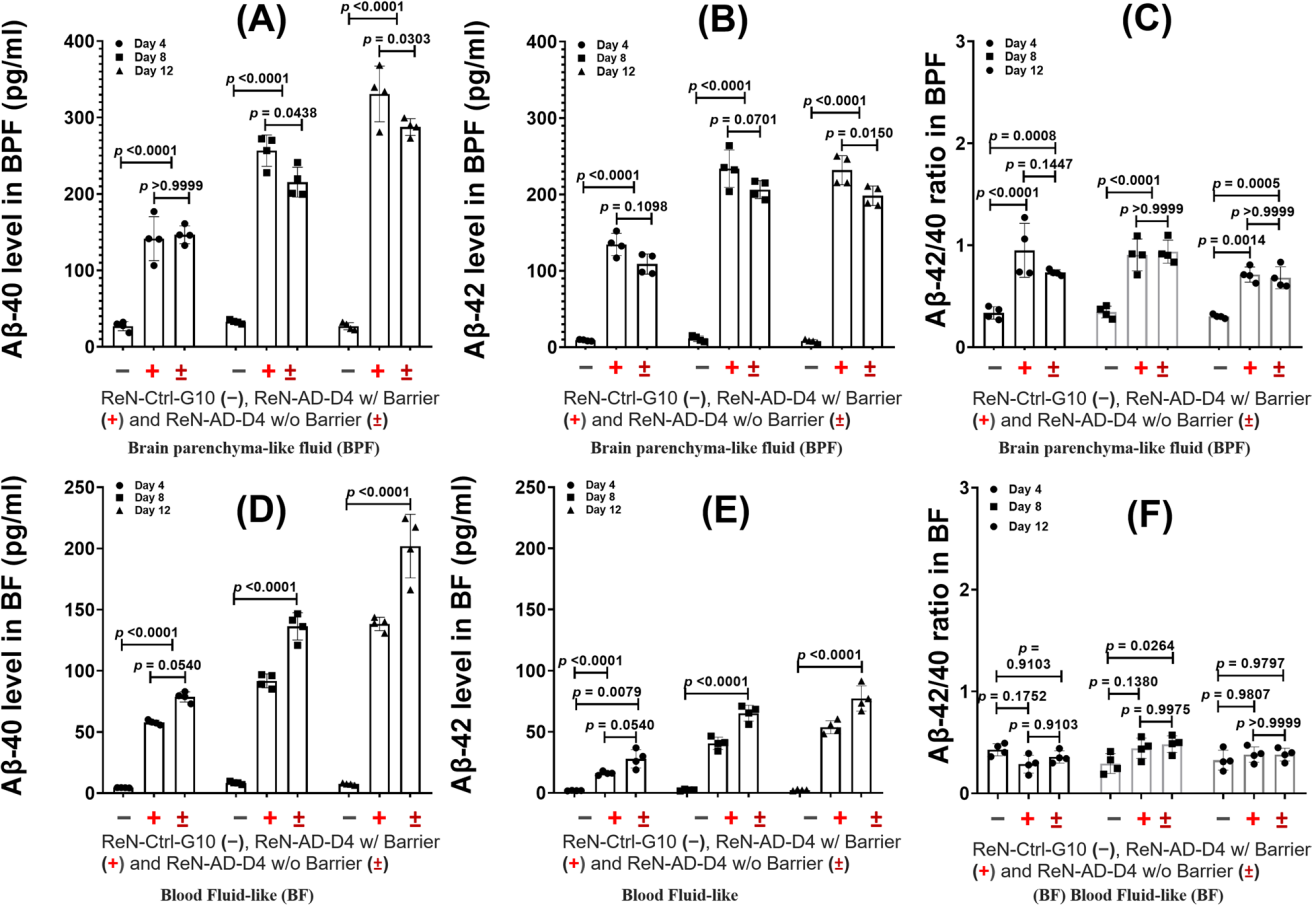
Secretion of Aβ40 and Aβ42 into brain parenchyma–like and blood-like compartments in neurospheroid-grafted microfluidic constructs. **A**, **D** Quantification of Aβ40 levels and **B**, **E** Aβ42 levels measured in media collected from the brain parenchyma–like fluid (BPF; **A–C** and blood-like fluid BF; **D**–**F** compartments of ReN-Ctrl-G10 and ReN-AD-D4 constructs. **C**, **F** Corresponding Aβ42/40 ratios calculated from matched samples. Data are presented as mean ± SD from *n* = 4 independent experiments, with four microfluidic chips analyzed per condition. Statistical analysis was performed using two-way ANOVA with post hoc multiple-comparisons testing. Exact *P* values for each comparison are reported directly on the graphs

Aβ40 and Aβ42 secretion was quantified in neurospheroid-grafted endothelial barrier constructs over 4, 8, and 12 days using ELISA (Fig. 10). ReN-AD-D4 constructs showed markedly higher levels of both Aβ40 and Aβ42 in the brain parenchyma-like fluid (BPF) and blood-like fluid (BF) compared with ReN-Ctrl-G10 constructs.

Within neurospheroids, Aβ40 concentrations in ReN-Ctrl-G10 remained low and consistent over time, measuring 27.00 ± 0.70 pg/mL on day 4, 32.80 ± 0.40 pg/mL on day 8, and 26.90 ± 0.80 pg/mL on day 12. In contrast, ReN-AD-D4 neurospheroids showed progressively increasing Aβ40 levels of 141.40 ± 2.80 pg/mL on day 4, 256.70 ± 6.22 pg/mL on day 8, and 330.80 ± 5.33 pg/mL on day 12 (Fig. 10A). Aβ42 concentrations in ReN-Ctrl-G10 remained similarly low at 9.10 ± 1.10 pg/mL, 11.20 ± 3.00 pg/mL, and 8.10 ± 1.20 pg/mL on days 4, 8, and 12, respectively, whereas ReN-AD-D4 constructs showed elevated values of 134.50 ± 25.40 pg/mL on day 4 and 232.40 ± 1.30 pg/mL on days 8 and 12 (Fig. 10B).

In the BF, Aβ40 concentrations in ReN-AD-D4 constructs increased from 57.70 ± 0.60 pg/mL on day 4 to 91.70 ± 5.40 pg/mL on day 8 and 137.80 ± 1.60 pg/mL on day 12. Aβ42 levels similarly rose from 16.10 ± 0.10 pg/mL on day 4 to 40.40 ± 3.00 pg/mL on day 8 and 52.60 ± 2.50 pg/mL on day 12 (Figs. 10D–E). In ReN-AD-D4 constructs lacking an endothelial barrier, Aβ40 and Aβ42 concentrations were higher in the BF and lower in the BPF than in neurospheroid-grafted endothelial barrier constructs, reflecting differences in Aβ distribution in the presence or absence of an endothelial barrier (Fig. 10A–E).

Analysis of the Aβ42/Aβ40 ratio showed distinct patterns between compartments. In the BF, the ratio on day 4 was lower in ReN-AD-D4 neurospheroid-grafted endothelial barrier constructs (0.28 ± 0.0003) compared with ReN-AD-D4 constructs without a endothelial barrier (0.35 ± 0.0006) (Fig. 10F). In the BPF, relatively higher Aβ42/Aβ40 ratios were observed, indicating greater retention of Aβ42 compared to Aβ40 within the neurospheroid compartment (Fig. 10C). No marked ratio differences were observed on days 8 and 12, consistent with the increased permeability measured at later time points (Fig. 5).

## Discussion

This study presents a microfluidic neurospheroid-grafted endothelial barrier model that captures key AD-associated neurovascular features. By integrating neurospheroids with a perfusable endothelial vessel and sustaining both tissues under gravity-driven bidirectional flow, the platform maintains physiologically relevant fluid exchange and shear forces without external pumps or tubing [26, 32]. The generated shear stress (0.5–5 dyne/cm^2^) falls within the physiological range required for endothelial alignment, tight-junction maintenance, and vascular remodeling, and is compatible with neuronal differentiation and network formation [26, 32, 35]. This flow regime, combined with the separated brain-like and vascular compartments, facilitates simultaneous analysis of Aβ distribution, endothelial barrier integrity, and neurovascular interactions without implying BBB-specific transport mechanisms.

This design enables the formation of a simple, perfusable endothelial vessel within the microfluidic perfusion channel, simplifies media sampling from both compartments, and minimizes cross-interference of secreted molecules, allowing quantitative analyses of Aβ distribution and barrier function. The model captures key AD features, including increased phosphorylated tau (pTau), Aβ accumulation, and endothelial barrier dysfunction, and thereby supports spatial analysis of neurovascular interactions. To support neurospheroid stability within the brain chamber, we incorporated a hydrogel-based extracellular matrix. Collagen I was selected because it preserves neurospheroid integrity, provides reproducible mechanical properties, and is compatible with perfusion-based microfluidic culture, enabling robust matrix patterning and long-term construct stability [36, 37]. We acknowledge that collagen I does not recapitulate the biochemical composition or stiffness of native brain parenchyma or the vascular basement membrane, and that matrix-dependent processes such as laminin/collagen IV–mediated endothelial polarization, integrin-specific signaling, and pericyte–endothelial crosstalk is therefore not fully modeled and should not be overinterpreted.

Although collagen I is not a native component of the brain extracellular matrix, it offers practical advantages over collagen IV, which more closely resembles the basement membrane [38] but did not maintain compartmental integrity in our hands [39]. Matrigel and fibrin-based matrices have previously been used in tri-culture BBB models. They can support localized Aβ deposition [40], but in our system, they promoted excessive neurite outgrowth and impaired barrier performance. Accordingly, a collagen I–based matrix provided the optimal balance of structural stability and compatibility with dynamic neurovascular interactions, while explicitly prioritizing reproducibility and long-term perfusion over basement-membrane–specific signaling fidelity within this endothelial barrier system.

Within this collagen I matrix, AD-mutant neurospheroids exhibited a 7.6-fold increase in pTau accompanied by heightened endothelial barrier permeability and reduced ZO-1 expression, reflecting co-occurring neuronal and endothelial alterations. MAP2 expression presented a downward trend in the ReN-AD-D4 model, suggesting early neuronal cytoskeletal disruption even before overt loss of neuronal markers. Hyperphosphorylated tau can sequester MAP2, impair microtubule assembly, and destabilize dendritic structures, leading to synaptic dysfunction and neurite degeneration [41]. Loss of MAP2 compromises cytoskeletal stability and neurite integrity, potentially exacerbating neurodegenerative processes.

Although elevated pTau and endothelial barrier disruption emerged in parallel within the construct, the present data do not establish mechanistic linkage between neuronal tau pathology and vascular impairment. Instead, these observations highlight the platform’s ability to capture multiple AD-associated phenotypes within a shared microenvironment [42]. Supplementary analyses of neurospheroids in Matrigel further confirmed AD-associated marker expression, but the primary functional studies presented here focus on collagen I, which provided superior structural stability. Future work targeting tau phosphorylation and MAP2 preservation will be important for determining whether tau-related cytoskeletal changes contribute directly or indirectly to endothelial barrier compromise in this microfluidic system [43, 44].

Aβ exposure imposed substantial structural stress on the endothelial barrier, producing fragmented F-actin networks and discontinuous ZO-1 and CD31 labeling. These findings align with prior reports showing that Aβ disrupts cytoskeletal organization and weakens tight junctions [45, 46]. Quantitative CTCF measurements confirmed significant reductions across all three markers, and integration of these structural readouts with permeability data using a Barrier Integrity Index (BII) revealed an order-of-magnitude decrease in endothelial barrier integrity, indicating that cytoskeletal deterioration and increased permeability emerge concurrently in this model. Functionally, these structural deficits corresponded with increased permeability and reduced ZO-1 expression in the neurospheroid-grafted constructs, consistent with prior in vitro and in vivo observations of Aβ-associated endothelial barrier disruption [31, 40]. Aβ has been shown to destabilize junctional complexes including ZO-1, occludin, and claudin-5, promoting paracellular transport and progressive barrier weakening [42, 47–50]. Although P_app_ values in our model were modestly higher than typical in vivo estimates, this is expected for simplified endothelial systems and reflects differences in tight-junction density, matrix composition, and microfluidic flow characteristics [51–53].

ELISA measurements provided additional insight into the dynamics of soluble Aβ. Initially, the endothelial barrier retained both Aβ42 and Aβ40 within the brain parenchyma-like compartment, indicating early barrier integrity [54]. Over time, however, Aβ levels rose in both the brain parenchyma-like fluid and the blood-like perfusate, consistent with increased Aβ burden within the construct rather than BBB-specific transport dynamics. These changes may reflect generalized endothelial stress and loss of junctional continuity associated with increasing amyloid burden, rather than engagement of specific endothelial transport or clearance mechanisms, which were not directly assessed in this study [55]. Immunostaining suggested that a portion of Aβ accumulates at or near endothelial junctions, in agreement with reports that Aβ can associate with tight-junction regions and accompany structural weakening [56]. Higher-resolution imaging and protein co-localization studies will be necessary to confirm the spatial relationship between Aβ aggregates and junctional proteins in this setting [57]. Evaluating inflammatory mediators such as TNF-α and IL-1β [58], together with regulators of Aβ handling, including LRP1 and RAGE [59], may provide deeper mechanistic insights.

The evolving Aβ42/40 ratios further reflected differential accumulation patterns of Aβ species over time. Early reductions in circulating Aβ42/40 ratios, accompanied by increased Aβ42 retention within the neurospheroid compartment, closely resemble clinical observations in AD patients [60]. These findings suggest that Aβ42 may be retained or deposited more readily within the brain-like environment compared with Aβ40, which appeared at higher levels in the perfusate indicating selective barrier properties. At later time points, the divergence in Aβ42/40 ratios diminished, corresponding with increased endothelial barrier permeability likely reflecting severe endothelial barrier disruption and loss of selective barrier properties. Extended Aβ variants, particularly Aβ42, exhibit higher aggregation propensity and form plaques in AD brain tissue [61], whereas Aβ40 preferentially accumulates in vascular regions and is relatively less neurotoxic [62]. In combination with the observed deposition of Aβ along the endothelial interface, these patterns align with features associated with cerebral amyloid angiopathy (CAA) although the present platform does not fully model the cellular complexity or vascular specialization of in vivo CAA. Nonetheless, the system provides a controlled environment to explore how differential Aβ species influence endothelial structure and function within a perfused microfluidic context.

While the model effectively captures AD-associated endothelial and neurovascular-like alterations, including Aβ accumulation at the endothelial barrier, reduced ZO-1 expression, and increased permeability, it employs a simplified endothelial interface composed solely of HUVECs. HUVECs are widely used in early-stage endothelial barrier or BBB-approximation models because they are accessible and respond robustly to shear stress [63], but they lack several BBB-specific transporters such as LRP1 that mediate Aβ clearance [55]. Reported permeability values for in vitro endothelial barriers span several orders of magnitude, with HUVEC-based models typically showing the highest permeability (approximately 10⁻⁵ cm/s), primary human brain microvascular endothelial cells displaying intermediate values (approximately 10⁻⁶ cm/s), and induced pluripotent stem cell–derived endothelial cells exhibiting the lowest permeability (approximately 10⁻⁷ cm/s) [64]. These differences highlight the need for more specialized endothelial sources. The absence of pericytes, astrocytes, microglia, and oligodendrocytes also limit the model’s capacity to fully recapitulate neurovascular unit complexity. Future studies will incorporate iPSC-derived brain endothelial cells together with additional neurovascular unit components to better model cellular cross-talk, Aβ processing, and BBB-related function [40]. With these refinements, the neurospheroid-grafted BBB platform presented here may serve as a powerful tool for studying AD-associated neurovascular features, evaluating therapeutic candidates, and advancing precision medicine in a scalable microfluidic format without implying full BBB recapitulation.

## Conclusions

This study establishes a neurospheroid-grafted, membrane-free endothelial barrier microfluidic model that captures key AD-associated neurovascular-like features. By combining FAD neurospheroids with a perfusable endothelial vessel under physiologically relevant shear, the platform enabled simultaneous investigation of Aβ generation, distribution, accumulation, and barrier disruption within a unified 3D microenvironment. The model reproduced AD-associated endothelial alterations, including increased permeability, tight-junction destabilization, endothelial cytoskeletal disorganization, and Aβ deposition along the barrier, and demonstrated impaired neurite outgrowth associated with AD-specific pathology.

These results highlight the utility of the system for studying neurovascular interactions relevant to AD and for evaluating therapeutic strategies aimed at restoring endothelial barrier integrity or modulating Aβ dynamics. Future incorporation of iPSC-derived brain endothelial cells and additional neurovascular unit components is expected to enhance biological specificity and broaden mechanistic insight into Aβ handling and BBB-related pathways. Overall, this pump-free, shear-controlled microfluidic platform represents a robust and scalable tool for disease modeling, drug screening, and tissue-engineering research focused on vascularized neural constructs.

## Supplementary Information

Below is the link to the electronic supplementary material.

Supplementary Material 1

## Data Availability

The data generated and analyzed during this study are available from the corresponding author upon reasonable request.
