# Supplementary material for: Recapitulating Alzheimer’s disease pathophysiology with a microfluidic neurospheroid-grafted endothelial barrier model
Source: Mol Brain. 2026 Mar 3;19:25. doi: 10.1186/s13041-026-01285-3 (PMC13077919; doi:10.1186/s13041-026-01285-3)
Supplement: Supplementary file 1 — Supplementary Material 1 [file 13041_2026_1285_MOESM1_ESM.docx]

**Supplementary Material**

**Strategies for Optimizing Neurospheroid Size**

**Figure S1. Effect of initial cell seeding number on spheroid size.** **(A)** Representative brightfield images of ReN-Ctrl-G10 spheroids generated using increasing initial cell seeding numbers (1 × 10⁴–5 × 10⁴ cells/well) at Day 14 of culture. **(B)** Representative brightfield images of ReN-AD-D4 spheroids formed under identical seeding conditions at Day 14. Scale bar = 300 μm. **(C)** Quantification of ReN-Ctrl-G10 spheroid diameter as a function of initial cell seeding number. **(D)** Quantification of ReN-AD-D4 spheroid diameter across the same seeding numbers. Bars represent mean ± SD from *n* = 2 independent experiments, with dots indicating individual experimental means (each dot represents the average diameter of 8 spheroids per experiment). Statistical significance was assessed by one-way ANOVA followed by post hoc comparisons of each higher seeding number relative to 1 × 10⁴ cells/well.

**AD-specific phenotypic expression of the Neurospheroid in Matrigel in the microfluidic device**


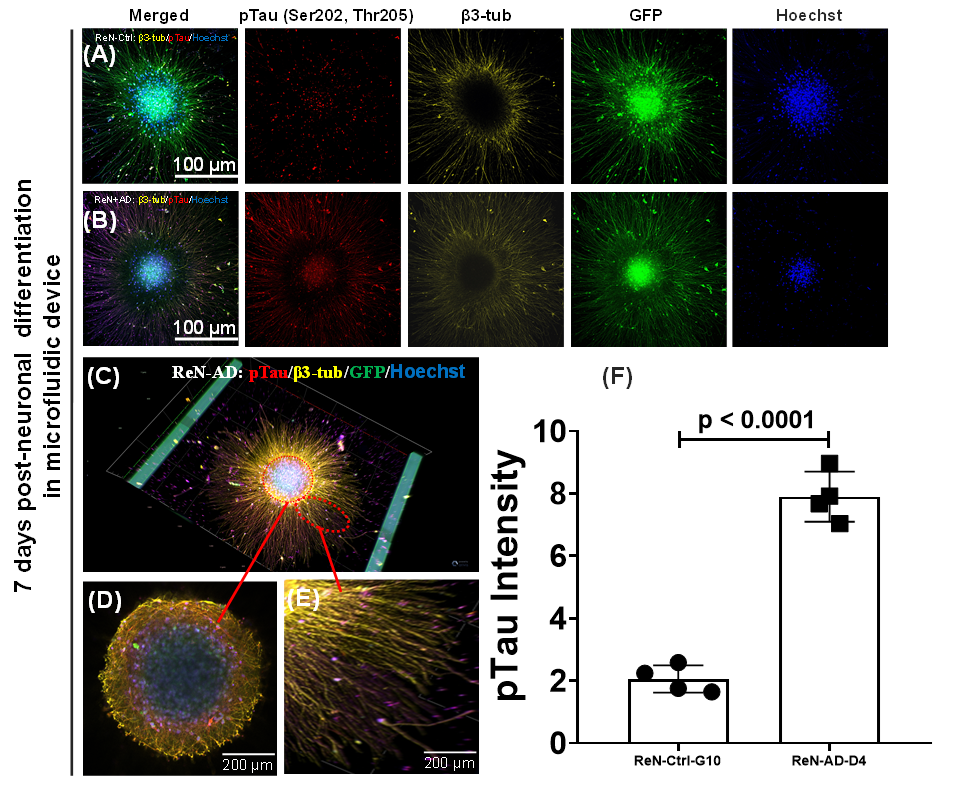


**Figure S2. Immunocytochemical characterization of differentiated neurospheroids in a microfluidic device.** **(A–B)** Representative confocal images of ReN-Ctrl-G10 **(A)** and ReN-AD-D4 **(B)** neurospheroids after 7 days of post-differentiation in a microfluidic device. Immunostaining shows phosphorylated tau (pTau, red), β3-tubulin (yellow; neuronal marker), GFP (green), and Hoechst (blue; nuclei). Both control and AD neurospheroids exhibit neuronal differentiation, with enhanced pTau signal in ReN-AD-D4 constructs. Scale bar = 100 μm. **(C)** Three-dimensional reconstruction of a confocal z-stack (10× objective) illustrating the spatial organization of pTau-positive neurons and neurite projections within the microfluidic chamber. **(D–E)** Higher-magnification views highlighting the neurospheroid cell mass **(D)** and radially extending neurites **(E)** expressing pTau (red) and β3-tubulin (yellow). Scale bar = 200 μm. **(F)** Quantification of pTau expression in ReN-Ctrl-G10 and ReN-AD-D4 neurospheroids. Data represent mean ± SD from n = 4 independent experiments, with 4 technical replicates per condition. Statistical significance was determined using an unpaired two-tailed t-test with Welch’s correction; p < 0.0001 relative to ReN-Ctrl-G





**Figure S3.** Characterization of neuronal and astrocyte differentiation markers in neurospheroids. **(A-B)** is the characterization of 7 days post-differentiated ReN-Ctrl-G10 and ReN-AD-D4 neurospheroids in the microfluidic device. Immunostaining result showed that after 7 days of post-differentiation on a microfluidic device, ReN cells started to express GFAP (red), an indirect marker for astrocytes, and MAP2 (yellow) which is the neuronal marker, respectively.
